# Putting Rigorous Evidence Within Reach: Lessons Learned from the New Heights Evaluation

**DOI:** 10.1007/s10995-020-02901-x

**Published:** 2020-03-24

**Authors:** Susan Zief, John Deke, Ruth Neild

**Affiliations:** Mathematica Policy Research, 600 Alexander Park, Princeton, NJ 08540 USA

**Keywords:** Teen parenting, Teen pregnancy, High school graduation, Program evaluation, Administrative data

## Abstract

**Purpose:**

This article uses an evaluation of New Heights, a school-based program for pregnant and parenting teens in the District of Columbia Public Schools, to illustrate how maternal and child health programs can obtain rigorous evaluations at reasonable cost using extant administrative data. The key purpose of the article is to draw out lessons learned about planning and conducting this type of evaluation, including the important role of partnerships between program staff and evaluators.

**Description:**

This article summarizes the evaluation’s research design, data sources, and lessons learned about ingredients contributing to the successful implementation of this study. The evaluation employed a difference-in-differences design to estimate program impacts using administrative data merged across agencies.

**Assessment:**

Several features of New Heights and its context facilitated an evaluation. First, New Heights leaders could clearly describe program components and how the program was expected to improve specific student education outcomes. These outcomes were easy to measure for program and comparison groups using administrative data, which agencies were willing to provide. Second, buy-in from program staff facilitated study approval, data agreements, and unanticipated opportunities to learn about program implementation. Finally, time spent by evaluators and program staff in conversation about the program’s components, context, and data resulted in greater understanding and a more useful evaluation.

**Conclusion:**

The New Heights evaluation is a concrete example of how a small program with a modest evaluation budget can obtain evidence of impact. Collaborative relationships between researchers and program staff can enable these informative studies to flourish.

## Significance

Programs that seek to improve outcomes for parents and children are increasingly asked to provide rigorous evidence of their effectiveness. However, programs without large budgets for evaluation can be daunted by the apparent challenges of building rigorous evidence about the effectiveness of their approach. Identifying an appropriate comparison group and obtaining data at reasonable cost are two of the biggest hurdles. The good news is that there are alternative evaluation designs that do not involve expensive sample recruitment or primary data collection.

## Introduction

Programs that seek to improve outcomes for parents and children are increasingly asked to provide rigorous evidence of their effectiveness. Typically, programs must provide evidence that outcomes for program participants improved relative to a comparison group that did not experience the program. Funders, including government agencies and private philanthropy, may want this evidence to understand the impact of their investment or to justify additional support for a program. Information about program effectiveness, coupled with an analysis of factors that support or impede implementation, is an essential ingredient for an evidence-informed approach to program development and improvement.

However, programs without large budgets for evaluation can be daunted by the apparent challenges of building rigorous evidence about the effectiveness of their approach. Identifying an appropriate comparison group and obtaining data at reasonable cost are two of the biggest hurdles. Seeking evidence of effectiveness, some programs focus their limited evaluation budgets on primary data collection, which is typically one of the key cost drivers in an evaluation. When their budgets cannot accommodate much data collection, some programs settle for studies with samples that are too small to detect impacts even if the program were effective. The good news is that there are alternative evaluation designs that do not involve expensive sample recruitment or primary data collection. In some cases, extant data from multiple agencies can be used creatively to design informative, high quality effectiveness evaluations at lower cost. Data from local health departments and school districts, for example, can provide a wealth of information to evaluate program success.

This article uses an evaluation of New Heights, a school-based program for pregnant and parenting teens in the District of Columbia Public Schools (DCPS), to illustrate how it can be an option for some programs to conduct a rigorous evaluation using extant administrative data. Although we briefly summarize the evaluation’s design and findings (reported in detail in Asheer 2017), the key purpose of this article is to draw out “lessons learned” about the process of planning and conducting this type of evaluation. These lessons can inform maternal and child health programs that are exploring options to evaluate program effectiveness—and demonstrate that rigorous evaluation might be within their reach.

## Description

### New Heights’ Purpose and Components

Although the teen birth rate reached a record low in 2017 of 18.8 births per 1000 females ages 15–19 (Martin et al. 2018), this rate translates into almost 200,000 American teenagers becoming mothers annually. These young mothers and their partners face daunting challenges in building stable and healthy lives for themselves and their children (Mollborn 2007; Brien and Willis 2008). Teen mothers have difficulty attending and completing high school, in part because they lack sufficient resources for housing, food, health services, and childcare (Maynard & Hoffman 2008). About half of teen mothers receive a high school diploma by age 22 (Perper et al. 2010).

In 2010, recognizing the difficulties faced by expectant and parenting teens, the Office of Population Affairs (formerly the Office of Adolescent Health) at the U.S. Department of Health and Human Services launched the Pregnancy Assistance Fund (PAF). The program seeks to improve intermediate outcomes, such as access to health care and education, which in turn are hypothesized to delay a subsequent pregnancy and bolster the long-term well-being of teen parents and their children.

In the 2011–2012 school year, DCPS used a PAF grant to expand an existing program, New Heights, to all of its large comprehensive high schools. New Heights is a voluntary, school-based program of supports to help expectant and parenting students—both mothers and fathers—navigate the challenges of pregnancy and parenthood and complete high school. Recognizing that expectant and parenting students can feel overburdened, embarrassed, and discouraged, the program seeks to reorient students toward identifying immediate education goals, making longer-term plans, and identifying clear pathways for achieving them.

New Heights’ key feature is a dedicated program coordinator in every school. Coordinators are trained staff employed by the district who deliver the program’s multiple components, tailoring their efforts to the needs of their students. Coordinators are responsible for integrating four main components into the regular school day: (1) advocacy; (2) targeted school-based case management; (3) weekly educational workshops; and (4) small rewards, primarily in the form of baby care products, for program participation. Taken together, these components aim to help expectant and parenting students identify their strengths and use them to overcome challenges to self-sufficiency and educational success.

To increase school engagement through improved attendance, New Heights supports students in overcoming the barriers, such as childcare and school policies, that keep them out of the classroom. The program also aims to increase credit accumulation and empower students to advocate for themselves. These short-term outcomes are expected to lead to long-term improvements such as increased graduation rates, postsecondary enrollment, employment, and the delay of subsequent pregnancies.

## The Evaluation Design

The evaluation used a difference-in-differences (DID) design to estimate the impact of New Heights for all eligible female students in study schools, including both program participants and non-participants. Although the program served both males and females, the study focused on females because they could systematically be identified as parenting and the males could not. The study focused on intermediate outcomes related to education but did not examine birth outcomes because the design required that outcomes be observable for all students, both parenting and non-parenting. Notably, the study used only retrospective, administrative data from DCPS, the District of Columbia Department of Health (DOH), and the District of Columbia Department of Human Services (DHS). Later in this article, we describe our strategies for obtaining and making sense of these data.

The DID study of the effectiveness of New Heights followed four main analytic steps:

1. For each outcome, the study team estimated the pre-post difference in relevant academic outcomes for parenting students. This estimate is the difference between outcomes for cohorts of students attending the schools *before* New Heights was introduced and outcomes for subsequent cohorts of students attending the same schools *after* the program was introduced.
2. To account for factors other than the New Heights program that might affect outcomes, the study team estimated the difference for non-parenting students attending the schools *before* the program was introduced and *after* the program was introduced.
3. To create a final estimate of program impact, the study team subtracted the non-parenting students’ pre-post difference (step 2) from the parenting females’ pre-post difference (step 1). This estimate is the “difference in differences” from which the method takes its name.
4. To refine the calculation of the impact for students who actually received services from New Heights (75% of those who were eligible), we divided the estimated impact for all eligible students by the proportion of eligible students who participated in New Heights.

**Fig. 1:**
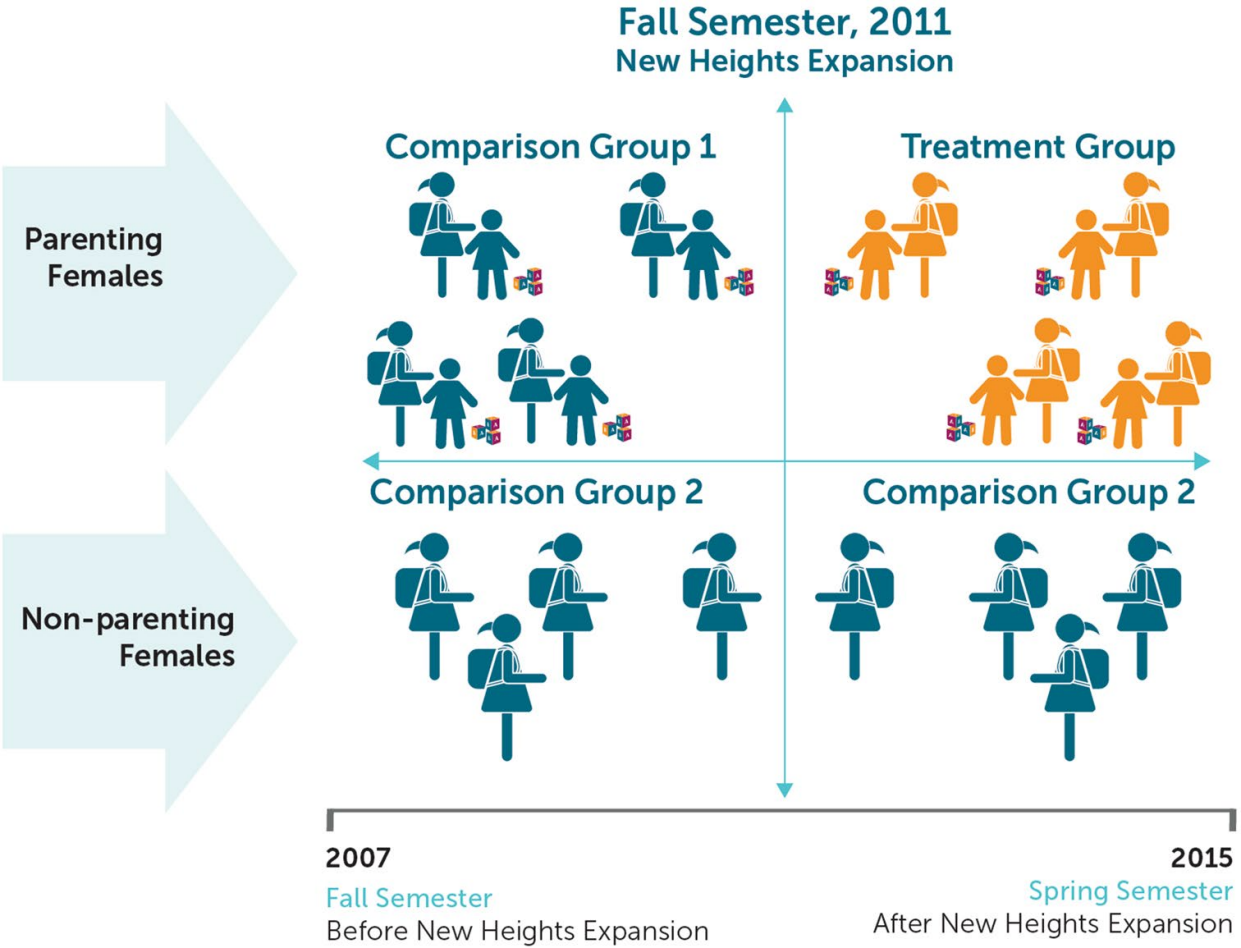
The impact analysis used two comparison groups

The DID approach was a good choice for this evaluation because the New Heights program operated in all DC comprehensive high schools during the study years. The program’s full coverage across comprehensive high schools meant that the study could not compare outcomes for students in the same cohort at schools with and without the program. Instead, the study compared outcomes for parenting students who had the opportunity to participate in New Heights to outcomes from previous cohorts of parenting students at the same schools (comparison group 1 in Fig. 1). To help rule out competing explanations for any impacts observed among parenting students, the study examined changes in outcomes for non-parenting students (comparison group 2 in Fig. 1). Examining changes for non-parenting students is a test of whether a factor other than the program—perhaps a new curriculum, better guidance, or stronger school leadership—could have affected outcomes for all students in the schools.

This design does not provide evidence as strong as that from a randomized controlled trial. Although we know of no applicable federal evidence clearinghouses that have standards for DID designs like this one, the DID approach used here does have an important advantage relative to the typical matched comparison design: it avoids bias due to unobserved differences between the types of parenting youth who choose to participate in New Heights and those who do not. The study accomplishes this by including in the treatment group all parenting youth, not just those who choose to participate, a strategy that is analogous to calculating an intent-to-treat impact in an RCT.

The study was reviewed and approved by the Institutional Review Boards of the relevant agencies whose data were used in the evaluation. The data for this study are extant administrative data captured retrospectively. The Institutional Review Boards for the agencies did not require consent to be collected for individuals for the purpose of this study.

### What the Evaluation Found About Program Effectiveness

Using the DID approach described above, the evaluation found that the New Heights program had positive effects on all outcomes examined. The program reduced the number of unexcused absences by 4.5 days compared to the average for parenting students who attended the schools prior to New Heights (a decrease of about 18.6%) and increased credit accumulation by 1.1 credits (a 24.4% increase). Among students who were 17 years and older, New Heights increased the graduation rate among by three percentage points (an 18.8% increase compared to the rate for parenting students prior to New Heights).

## Assessment

### Lessons Learned: Key Features that Facilitated the Evaluation

Several features of the New Heights program and its context facilitated an impact evaluation, as we describe in depth in the following sections. The program had a well-specified logic model and access to administrative data on births, program participation, and the education outcomes it hoped to improve. Program leaders were deeply confident in their approach and welcomed an independent evaluation, and agency researchers helped the evaluators to access and understand the administrative data. Even so, not all evaluation conditions for New Heights were ideal. The evaluation began four years after the program began to offer services, which ruled out some design options. The encouraging lesson of New Heights, however, is that obtaining rigorous evidence of effectiveness does not necessarily require optimal conditions.

### New Heights Had a Well-Specified Logic Model that Included Specific, Measurable Outcomes

Logic models are the cornerstone of a strong evaluation of program effectiveness. A good logic model specifies the key components of a program that are hypothesized to produce one or more specific outcomes. New Heights leaders were able to clearly describe the components of their program; how those components fit together; and how the program was expected to improve specific, meaningful education outcomes (attendance, course credits, and graduation). These outcomes were easy to measure for the program and comparison groups using administrative data.

Further, New Heights was a mature program that had operated for several years and had honed its vision. It had excellent staff monitoring and supervision to ensure that the shared vision was implemented. All of the work to develop and refine the program made New Heights ready for an impact evaluation.

### Administrative Data Were Available and Could Be Merged Across Agencies

The study relied on merged, longitudinal administrative data from DCPS, DOH, and DHS for 2007–2015. These data were initially collected for purposes other than evaluation (such as basic recordkeeping and compliance reporting) but could be used in new ways by the evaluation team to learn about the impact of the program. Using administrative data—rather than surveys or interviews—also reduced evaluation costs and the data collection burden on program staff and participants.

To obtain these data, the evaluators executed separate data agreements with each agency. Because DCPS and DHS were already collaborating on the program’s implementation, both agencies were invested in the success of the evaluation and sought to support the evaluation team through the agencies’ research review processes.

The study used data from 2007 through 2015 to:

*Identify females in the schools with and without children.* Data from DOH identified teens who had given birth in the district and the date(s) they gave birth.

*Form comparison groups.* Administrative data from DCPS, DOH, and DHS enabled the study to create a comparison group of parenting students who attended the schools prior to the introduction of New Heights and a second comparison group composed of non-parenting students.

*Assess outcomes.* Key education outcomes—attendance, credit accumulation, and graduation—were standard information kept by the school district for all high school students across multiple years.

*Assess program participation.* DHS records identified parenting students in the study schools who also participated in New Heights.

The evaluators merged DCPS and DOH data using students’ first name, last name, and birth date. Although some student data could be matched on exact name and date of birth, other matches could be made only by using an approximate spelling of the first and/or last name. To assess the quality of matches, the team hand-reviewed a randomly selected set of cases for accuracy. This in-depth examination of match quality showed that some matching strategies were acceptable (93% accuracy or more), but other strategies could not be used because they produced too many inaccurate matches.

### Buy-in from Program Staff Was the “Magic Ingredient” that Enabled the Evaluation to Move Forward Smoothly

From the start, New Heights program leadership welcomed the evaluation. Because they had been tracking their results through performance data, program staff had a data-informed confidence that their program was making a difference for teen parents. Still, the program staff needed time to develop confidence in the evaluation team and become convinced that the study design was the most rigorous, unbiased, and best possible for demonstrating program effectiveness. Once program staff understood the design and the evidence that it could provide, they were willing to champion the evaluation within DCPS, DHS, and DOH so that evaluators could acquire the necessary data.

Although a program implementation study had not been planned initially, the growing trust and regular communication between program staff and evaluators enabled them to put an implementation study in place when the opportunity arose. These qualitative research activities, which were ongoing while administrative data were being collected, cleaned, and analyzed, were invaluable in making sense of the impact findings. The impact and implementation findings enabled telling a story of not only whether the program had an impact but why it might have had that impact.

### Time Spent by Evaluators to Understand the Program’s Components, Context, and Data Paid Off in a More Useful Evaluation

As they sought to develop an evaluation plan, New Heights program staff and the study team held repeated, extensive conversations in person and via email about the program and the district context. Through these conversations, program staff and evaluators developed a design for a retrospective, rigorous evaluation that was feasible within the local context, time, and budget. One member of the evaluation team reported, “It was through the interaction between the evaluators and the program staff that the research design became something that we could imagine. We initially had modest expectations. But as we talked, the ideas began to flow.”

When the evaluation got underway, DCPS researchers and program staff spent time making sure that the evaluators understood the changing institutional context (such as changes in DCPS policies) to place findings in context. This work included discussions about changes in how administrative data elements were reported, coded, and calculated by DCPS across the eight years of data.

### How Programs Can Get Started with Evaluation

Although the use of administrative data substantially reduced the cost of the New Heights evaluation, the study did receive funding from the U.S. Department of Health and Human Services. Programs that do not have these resources could take some of the following steps to get ready to evaluate their effectiveness:

- Refine the program’s logic model until it faithfully represents what the program offers and the outcomes it expects to affect. Logic models are a basic building block of evaluation.
- Take time to learn about the administrative data that is available or might become available. The more that program staff learn about these data—including the data elements, the completeness and quality of the data, and the potential to crosswalk data across agencies—the better prepared they will be to provide this important information to a potential evaluator. This information can help them to assess the feasibility of an evaluation and develop an approach that can work for the program.
- Consider whether there are elements of the study design used for New Heights that can be copied outright or slightly adapted for the evaluation. There are no copyrights for research designs!
- Seek inexpensive sources of evaluation assistance, such as graduate students at local universities—or even universities outside of the area. If program staff are able to explain their logic model clearly and provide good detail about available administrative data, they have a good chance of capturing the interest of academic researchers.

## Conclusion

In recent years, there has been growing interest in how researchers and program staff can work in partnership to conduct more rigorous research to answer questions that are important for policy and practice. The idea behind these partnerships is that when individuals with a variety of expertise and desire to learn are around the table, the resulting research will be stronger and more relevant. The New Heights study is a concrete example of how that kind of partnership produced evidence of impact for a relatively small program with a modest budget for evaluation. The lessons learned from this study are that creative, cost-effective designs are possible—and also that collaborative relationships between researchers and program staff can enable informative studies to flourish.
